# Electronic Cigarette Use Among High School Students and Its Association With Cigarette Use And Smoking Cessation, North Carolina Youth Tobacco Surveys, 2011 and 2013

**DOI:** 10.5888/pcd13.150564

**Published:** 2016-08-04

**Authors:** Li-Ling Huang, Sarah D. Kowitt, Erin L. Sutfin, Tanha Patel, Leah M. Ranney, Adam O. Goldstein

**Affiliations:** Author Affiliations: Sarah D. Kowitt, Gillings School of Global Public Health, University of North Carolina, Chapel Hill, North Carolina; Erin L. Sutfin, Department of Social Sciences and Health Policy, Wake Forest School of Medicine, Winston-Salem, North Carolina; Tanha Patel, Tobacco Prevention and Control Branch, North Carolina Department of Health and Human Services, Raleigh, North Carolina; Leah M. Ranney, Adam O. Goldstein, Center for Regulatory Research on Tobacco Communication, Lineberger Comprehensive Cancer Center, Tobacco Prevention and Evaluation Program, Department of Family Medicine, School of Medicine, University of North Carolina, Chapel Hill, North Carolina.

## Abstract

**Introduction:**

Although adolescent cigarette use continues to decline in the United States, electronic cigarette (e‑cigarette) use among adolescents has escalated rapidly. This study assessed trends and patterns of e‑cigarette use and concurrent cigarette smoking and the relationships between e-cigarette use and smoking cessation intentions and behaviors among high school students in North Carolina.

**Methods:**

Data came from high school students who completed the school-based, cross-sectional North Carolina Youth Tobacco Survey in 2011 (n = 4,791) and 2013 (n = 4,092). This study assessed changes in prevalence of e-cigarette and cigarette use from 2011 through 2013, and cessation-related factors associated with those students’ current and past use of e‑cigarettes in 2013.

**Results:**

The prevalence of current e-cigarette use (use in the past 30 days) significantly increased from 1.7% (95% CI, 1.3%–2.2%) in 2011 to 7.7% (95% CI, 5.9%–10.0%) in 2013. Among dual users, current e-cigarette use was negatively associated with intention to quit cigarette smoking for good (relative risk ratio [RRR] = 0.51; 95% CI, 0.29–0.87) and with attempts to quit cigarette smoking in the past 12 months (RRR = 0.69; 95% CI, 0.49–0.97). Current e-cigarette smokers were less likely than those who only smoked cigarettes to have ever abstained from cigarette smoking for 6 months (RRR = 0.42; 95% CI, 0.21–0.82) or 1 year (RRR = 0.21; 95% CI, 0.09–0.51) and to have used any kind of aids for smoking cessation (RRR = 0.46; 95% CI, 0.29–0.74).

**Conclusion:**

Public health practitioners and cessation clinic service providers should educate adolescents about the risks of using any nicotine-containing products, including e-cigarettes, and provide adequate tobacco cessation resources and counseling to adolescent tobacco users.

**MEDSCAPE CME**

Medscape, LLC is pleased to provide online continuing medical education (CME) for this journal article, allowing clinicians the opportunity to earn CME credit.

This activity has been planned and implemented through the joint providership of Medscape, LLC and *Preventing Chronic Disease*. Medscape, LLC is accredited by the Accreditation Council for Continuing Medical Education (ACCME), the Accreditation Council for Pharmacy Education (ACPE), and the American Nurses Credentialing Center (ANCC), to provide continuing education for the healthcare team.

Medscape, LLC designates this Journal-based CME activity for a maximum of 1.00 ***AMA PRA Category 1 Credit(s)™***. Physicians should claim only the credit commensurate with the extent of their participation in the activity.

All other clinicians completing this activity will be issued a certificate of participation. To participate in this journal CME activity: (1) review the learning objectives and author disclosures; (2) study the education content; (3) take the post-test with a 75% minimum passing score and complete the evaluation at http://www.medscape.org/journal/pcd; (4) view/print certificate.

Release date: August 4, 2016; Expiration date: August 4, 2017.

**Learning Objectives**

Upon completion of this activity, participants will be able to:

1. Distinguish trends in the use of electronic cigarettes (e-cigarettes) and regular cigarettes among adolescents
2. Assess tobacco cessation strategies used by adolescents
3. Analyze variables associated with e-cigarette use among adolescents
4. Evaluate the relationship between e-cigarette use and tobacco cessation beliefs and behaviors among adolescents

**EDITOR**

Rosemarie Perrin

Editor, *Preventing Chronic Disease*

Disclosure: Rosemarie Perrin has disclosed no relevant financial relationships.

**CME AUTHOR**

Charles P. Vega, MD

Health Sciences Clinical Professor of Family Medicine, University of California, Irvine, California

Disclosure: Charles P. Vega, MD, has disclosed the following relevant financial relationships:

Served as an advisor or consultant for: Allergan, Inc.; McNeil Consumer Healthcare

Served as a speaker or a member of a speakers bureau for: Shire

**AUTHORS**

Li-Ling Huang, PhD

Center for Regulatory Research on Tobacco Communication, Lineberger Comprehensive Cancer Center, University of North Carolina, Chapel Hill, North Carolina

Disclosure: Li-Ling Huang, PhD, has disclosed no relevant financial relationships.

 Sarah Kowitt, MPH

Gillings School of Global Public Health, University of North Carolina, Chapel Hill, North Carolina

Disclosure: Sarah Kowitt, MPH, has disclosed no relevant financial relationships.

 Erin L. Sutfin, PhD

Department of Social Sciences and Health Policy, Wake Forest School of Medicine, Winston-Salem, North Carolina

Disclosure: Erin L. Sutfin, PhD, has disclosed no relevant financial relationships.

 Tanha Patel, MPH

Tobacco Prevention and Control Branch, North Carolina Department of Health and Human Services, Raleigh, North Carolina

Disclosure: Tanha Patel, MPH, has disclosed no relevant financial relationships.

 Leah Ranney, PhD

Center for Regulatory Research on Tobacco Communication, Lineberger Comprehensive Cancer Center, and the Tobacco Prevention and Evaluation Program, Department of Family Medicine, School of Medicine, University of North Carolina, Chapel Hill, North Carolina

Disclosure: Leah Ranney, PhD, has disclosed no relevant financial relationships.

 Adam O. Goldstein, MD, MPH

Center for Regulatory Research on Tobacco Communication, Lineberger Comprehensive Cancer Center, and the Tobacco Prevention and Evaluation Program, Department of Family Medicine, School of Medicine, University of North Carolina, Chapel Hill, North Carolina

Disclosure: Adam O. Goldstein, MD, MPH, has disclosed no relevant financial relationships.

## Introduction

Electronic cigarettes (e-cigarettes) are devices that aerosolize liquid that contains nicotine, humectants, and flavoring agents and mimic the experience of cigarette smoking. These devices are being aggressively marketed as smoking cessation aids and as healthy alternatives to cigarette smoking (1,2). Although e-cigarettes are not approved by the US Food and Drug Administration (FDA) as a cessation aid, they are perceived as healthier than cigarettes by adolescents (3), young adults (4), and adults (5,6). Adolescent cigarette smokers report that one reason they try e-cigarettes is because they want to quit cigarettes (3). But, unlike adults’ reasons for using e-cigarettes, adolescents’ top reason is not a desire to reduce cigarette smoking (6–10); for adolescents, curiosity, appealing flavors, and peer influences rank as higher reasons (3).

Public health concerns about adolescent e-cigarette use have been raised because the adolescent’s developing brain is particularly vulnerable to the negative effects of nicotine neurotoxin and nicotine dependence (11–13). Nicotine dependence may develop rapidly among at-risk youths who are still at relatively low levels of smoking, even before they progress to regular or daily smoking (14,15). Longitudinal evidence shows that early emerging dependence symptoms in adolescence predict a greater predisposition for continued smoking behavior in young adulthood (16). High school students who are exposed to nicotine in early adolescence are also at higher risk for becoming highly nicotine dependent than are those exposed to nicotine later in adolescence, leading to more difficulty quitting (15). Having a better understanding of the relationship between e-cigarette use and smoking cessation intentions and behaviors among adolescents can inform FDA regulatory efforts on adolescent e-cigarette use, including communicating harmful health effects of e-cigarette use to youths, correcting misperceptions about their role in smoking cessation and nicotine addiction, and providing adolescents with adequate cessation resources.

Little data exists on how adolescents use e-cigarettes in an attempt to quit cigarette smoking. Recent cross-sectional data show that e-cigarette use among US adolescents was associated with lower odds of abstinence from cigarette use for 30 days or more (17). Planning to quit smoking cigarettes within the next year was positively associated with ever using e-cigarettes, but not with currently using e-cigarettes, and attempts to quit smoking were not associated with ever using or currently using e-cigarettes (17). Another US study of adolescent smokers reported that ever using e-cigarettes was not associated with an intention to quit smoking (18). In a study of Korean adolescents, e-cigarette use was associated with higher odds of ever having attempted to quit smoking in the past 12 months among current cigarette smokers and lower odds of smoking cigarettes in the past 30 days among youths who had ever smoked cigarette, suggesting some youths may be using e-cigarettes as a cessation aid (19). These studies showed that current e-cigarette users were significantly less likely to have abstained from smoking cigarettes in the past 30 days; nevertheless, the relationships between e-cigarette use and intentions or attempts to quit cigarettes were mixed. Recent evidence supports the efficacy of psychosocial interventions for smoking cessation among adolescents, and some findings support the efficacy of nicotine replacement treatments (20). However, there is insufficient evidence to determine the efficacy of pharmacological treatments among adolescents (20).

We used cross-sectional data from the 2011 and 2013 North Carolina Youth Tobacco Survey (NCYTS) to examine relationships between using e-cigarettes and cigarette smoking among adolescents and to test whether e-cigarette use is associated with intention to quit cigarette smoking, attempts to quit, and various quit methods. In addition, we studied trends in adolescents’ e-cigarette use over time.

## Methods

### Data source

The NCYTS is a voluntary, anonymous, school-based survey of middle and high school students administered biannually since 1999. The NCYTS survey uses a 2-stage cluster probability sampling design to produce a representative sample of students in grades 6 through 12 (21). Our study consisted of public high school students only (grades 9–12) because adolescents of high school age are more vulnerable than younger adolescents to experimenting with risky behaviors, including dual use of cigarette and e-cigarettes, The Centers for Disease Control and Prevention funded and approved the NCYTS, which is conducted to evaluate state tobacco control efforts. Our study, which used secondary data analysis, was reviewed by the Office of Human Research Ethics at the University of North Carolina, Chapel Hill, which determined that the study did not constitute human subjects research as defined under federal regulations 45 CFR 46.102 (d or f) and 21 CFR 56.102(c)(e)(l) and did not require institutional review board approval.

### Measurement

**Use of e-cigarettes and cigarettes. **To assess cigarette smoking, we asked students whether they had ever tried cigarette smoking, even 1 or 2 puffs; when the last time was they smoked a cigarette, even 1 or 2 puffs; and how many days they smoked cigarettes during the past 30 days. Students who reported that they had ever smoked cigarettes, but not in the past 30 days, were categorized as “past users.” Those who reported smoking cigarettes at least 1 day in the past 30 days were categorized as “current users.” Current and past use of e-cigarettes was assessed by 2 questions: “In the past 30 days, which of the following tobacco products have you used on at least one day?” and “Which of the following tobacco products have you ever tried, even just one time?” Students who responded they had used “electronic cigarettes or e-cigarettes, such as Ruyan or NJOY” on at least 1 of the past 30 days were categorized as current users. Those who said they had ever used e-cigarettes, but not in the past 30 days, were categorized as past users. Students who never tried cigarettes or e-cigarettes were categorized as “never users.” Students who both smoked cigarettes and used e-cigarettes in the past 30 days were categorized as current users of both. Students were also asked about their intention to try e‑cigarettes with the question (asked in the 2013 survey only), “In the next year, which of the following tobacco products do you think you will try?” Students who responded “electronic cigarettes or e-cigarettes, such as Ruyan or NJOY” were considered as intending to try e-cigarettes.

These demographic variables were used as covariates in analysis to adjust for sex, grade (9^th^–12^th^), and race/ethnicity (white, black, Hispanic, and other).

**Intention to quit cigarette smoking. **Intention to quit cigarette smoking was measured with the question “Do you want to stop smoking cigarettes for good?” with response options “Yes,” “No,” and “I do not smoke now.” Quit attempts for cigarette smoking were measured with the question “During the past 12 months, how many times have you stopped smoking for 1 day or longer because you were trying to quit smoking cigarettes for good?” Students who chose 1 or more times (possible responses were 1 time, 2 times, 3–5 times, 6–9 times, and ≥10 times) were categorized as having tried to quit smoking; those who chose “did not try to quit smoking cigarettes” were categorized as not having tried to quit smoking; those who reported not smoking cigarettes during the past 12 months were categorized as not smoking cigarettes. Length of last period of abstinence from cigarette smoking was based on responses to the question “When you last tried to quit for good, how long did you stay off cigarettes?” Response options were “less than 30 days,” “30 days,” “6 months,” and “1 year.”

Students were asked, “In the past 12 months, did you do any of the following to help you quit using tobacco of any kind for good?” Students could select 1 or more of the following responses (Table 1): “attended a program at my school,” “attended a program in the community,” “called a telephone help line or telephone quit line,” “used nicotine gum,” “used nicotine patch,” “used any medicine to help quit,” “visited an Internet quit site,” “got help from family or friends,” “used another method such as hypnosis or acupuncture,” “tried to quit on my own or quit cold turkey,” “I did not try to quit during the past 12 months,” and “I did not use tobacco of any kind during the past 12 months.” A variable was derived from answers to this question to classify students into 4 groups: “used any quit aid” for those who used any aid but not quit cold turkey, “quit cold turkey only” for those who answered “tried to quit on my own or quit cold turkey” only, “did not attempt to quit,” or “did not use tobacco.”

**Table 1:** Smoking Cessation Methods Attempted by Study Sample of Participants (N = 517)^a^ in Relation to E-Cigarette Use, 2013 North Carolina Youth Tobacco Survey

Cessation Method	Used E-Cigarettes?			
	Total^a^ N (%)^b^	Never (n = 3,405), n (%)	In Past (n = 397), n (%)	Currently (n = 290), n (%)
Attended a program at my school	33 (6.6)	23 (66.5)	6 (20.0)	4 (13.5)
Attended a program in the community	36 (6.9)	32 (90.2)	3 (5.7)	1 (4.1)
Called a telephone help line or telephone quit line	22 (3.8)	18 (85.2)	2 (5.7)	2 (9.1)
Used nicotine gum	39 (8.7)	22 (62.4)	8 (18.7)	9 (18.9)
Used nicotine patch	22 (3.8)	16 (73.7)	1 (7.5)	5 (18.8)
Used any medicine to help quit	10 (1.7)	4 (42.5)	2 (10.0)	4 (47.5)
Visited an Internet quit site	6 (1.1)	2 (32.5)	2 (27.6)	2 (40.0)
Got help from family or friends	50 (8.3)	22(46.2)	16 (27.8)	12 (26.0)
Used another method such as hypnosis or acupuncture	4 (0.5)	3 (84.4)	1 (15.6)	0 (0.0)
Tried to quit on my own or quit cold turkey	357 (70.1)	163 (42.9)	106 (32.1)	88 (25.1)

a Study participants could choose all quit methods tried; therefore, number of responses (579) exceeds number of study respondents (517).

b The percentages are weighted data based on the total of 517 participants.

### Statistical analysis

NCYTS data are statistically weighted to reflect the likelihood of sampling each student and to reduce bias by compensating for differing patterns of nonresponse. Data were analyzed by using STATA version 13.1 (STATA Corp) survey procedures to account for the complex survey design and sampling weights unless stated otherwise. We used χ^2 ^tests to compare sample characteristics and to examine descriptive statistics for each covariate. Multinomial logistic regression analyses were conducted to examine associations between predictors (ie, cigarette use, quit intention, quit attempt, length of last abstinence period) and use of 3 outcome categories: never, past, and current e-cigarette use in 2013. Adjusted relative risk ratios (RRRs) were calculated in reference to the base group (ie, never e-cigarette users). Separate models were used for each cessation-related predictor because of collinearity between the predictors. All models were adjusted for sociodemographic variables.

## Results

**E-cigarette prevalence.** Participants in the current study were 4,791 students from 90 high schools who participated in the 2011 NCYTS and 4,092 students from 83 high schools who participated in the 2013 NCYTS. The overall response rates combining school and student levels were 78.2% in 2011 and 67.8% in 2013. Of the high school students in the 2011 and 2013 surveys, about half were male and more than 50% were non-Hispanic white (Table 2). The prevalence of current e-cigarette use among North Carolina high school students increased significantly, from 1.7% (95% CI, 1.3%–2.2%) in 2011 to 7.7 (95% CI, 5.9%–10.0%) in 2013 while current cigarette use declined from 15.1% (95% CI, 13.7%–16.7%) to 13.1% (95% CI, 11.6%–14.7%) (Table 2). A notable increase occurred in the proportion of current e-cigarette users who had never smoked cigarettes from 2011 (n = 7, 7.8%; 95% CI, 6.6%–8.6%) to 2013 (n = 26, 11.6%; 95% CI, 8.3%–16.0%). Among students who reported in the 2013 survey that they thought they would try e-cigarettes in the next year, 20% had never smoked cigarettes. Concurrent use of cigarettes and e-cigarettes was 4.4% (95% CI, 3.3–5.8) among students surveyed in 2013, which was 3 times more than concurrent use of both products in 2011 (1.3%; 95% CI, 1.0%–1.8%). Multinomial logistic regression analysis for both 2011 and 2013 confirmed that current cigarette use was strongly associated with both current and past e-cigarette use after adjusting for sociodemographic variables (RRR=18.68; 95% CI, 12.95–26.93 for current e-cigarette use in 2013; RRR = 46.17; 95% CI, 28.98–73.55 for past e-cigarette use in 2013) (Table 3).

**Table 2:** Sociodemographic Characteristics of Participants in the 2011 (N = 4,791) and 2013 (N = 4,092) North Carolina Youth Tobacco Survey, by E-Cigarette Use

Sample Characteristics	2011				2013			
	Full Sample (n = 4,791), n (% [95 % CI])	E-Cigarette User			Full Sample (n = 4,092), n (% [95 % CI])	E-Cigarette User		
		Never (n = 4,492), n (% [95 % CI])	Past (n = 204), n (% [95 % CI])	Current (n = 95), n (% [95 % CI])		Never (n = 3,405), n (% [95 % CI])	Past (n = 397), n (% [95 % CI])	Current (n = 290) n (% [95 % CI])
Smoked cigarettes?	4,791 (100.0)	4,492 (94.1 [93.0–95.0])	204 (4.2 [3.5–5.1])	95 (1.7 [1.3–2.2])	4,092 (100.0)	3,405 (82.2 [78.0–85.7])	397 (10.1 [8.2–12.5])	290 (7.7 [5.9–10.0])
Never	2,878 (61.3 [58.2–64.3])	2,863 (64.7 [61.8–67.5])	8 (5.8 [2.3–14.0])	7 (7.8 [6.6–8.6])	2,544 (63.2 [61.2–65.2])	2,449 (73.5 [70.5–76.3])	69 (18.5 [13.6–24.7])	26 (11.6 [8.3–16.0])
In the past	1,149 (23.6, 21.6–25.8])	1,060 (23.2, 20.9–25.7])	73 (36.4 [26.7–47.4])	16 (14.6 [8.0–25.2])	1,007 (23.8 [22.2–25.4])	716 (19.9 [18.2–21.9])	197(49.0 [42.7–55.4])	94 (31.1 [26.4–36.2])
Currently	764 (15.1 [13.7–16.7])	569 (12.1 [10.9–13.5])	123 (57.8 [47.2–67.8])	72 (77.6 [65.8–86.1])	541 (13.1, 11.6–14.7])	240 (6.5 [5.3–8.1 ])	131 (32.5 [27.4–38.0])	170 (57.3 [51.0–63.3])
**Sex**								
Female	2,577 (49.3 [46.1–52.4])	2,461 (50.4 [47.0–53.7])	88 (35.1 [26.6–44.7])	28 (23.5 [16.2–32.9])	2,195 (48.9 [46.0–51.8])	1,909 (51.0 [48.0–54.1])	189 (47.8 [40.5–44.8])	97 (27.9 [20.9–36.1])
Male	2,211 (50.7 [47.6–53.9])	2,208 (49.6 [46.3–53.0])	116 (64.9 [55.3–73.4])	67 (76.5 [67.1–83.8])	1,893 (51.1 [48.2–54.0])	1,493 (49.0 [45.9–52.0])	207 (52.2 [44.8–59.5])	193 (72.1 [63.9–79.1])
**Race/ethnicity**								
Non-Hispanic black	1,063 (32.2, 25.3–40.0])	1,041 (33.5 [26.3–41.5])	17 (11.8 [6.2–21.3])	5 (10.2 [4.1–23.1])	1,132 (27.3 [23.9–31.0])	1,028 (30.2 [26.4–34.3])	67 (17.9 [13.4–23.5])	37 (8.8 [5.1–14.6])
Non-Hispanic white	2,880 (55.9 [48.3–63.2])	2,644 (54.5 [46.6–62.1])	162 (79.7 [70.0–87.9])	74 (73.8 [61.5–83.2])	2,113 (54.0 [48.6–59.4])	1,650 (50.1 [44.9–55.2])	258 (67.7 [61.9–73.1])	205 (78.1 [66.1–86.7])
Non-Hispanic other	232 (3.9 [3.1–4.9])	215 (3.9 [3.1–50.0])	11 (3.8 [1.7–8.0])	6 (4.5 [1.5–12.6])	224 (7.5 [5.4–10.3])	192 (8.1 [6.2–10.9])	19 (4.5 [1.9–10.2])	13 (4.7 [2.1–10.4])
Hispanic	597 (8.0 [7.0–9.2])	574 (8.1 [7.0–9.4])	13 (4.7 [2.1–9.8])	10 (11.5 [5.0–24.4])	607 (11.2 [9.2–13.5])	519 (11.6 [9.6–13.9])	53 (9.9 [7.3–13.2])	35 (8.4 [4.9–14.2])
**Grade**								
9th	1,463 (29.9 [23.3–37.4])	1,395 (30.7 [23.9–38.5])	46 (14.9 [9.2–23.3])	22 (18.9 [10.4–31.6])	1,193 (28.8 [22.4–36.2])	1,069 (31.5 [25.3–38.5])	72 (16.8 [11.1–24.6])	52 (15.4 [8.7–25.8])
10th	1,269 (26.0 [21.9–30.6])	1,201 (26.0 [21.8–30.7])	46 (28.4 [21.3–36.8])	22 (20.9 [12.6–32.6])	931 (25.9 [21.1–31.3])	790 (25.7 [21.4–30.5])	89 (30.0 [19.5–43.0])	52 (22.1 [14.3–32.5])
11th	1,081 (23.3 [19.0–28.3])	1,010 (23.2 [18.8–28.2])	53 (27.4 [18.4–38.6])	18 (23.2 [14.4–35.1])	957 (23.4, 19.0–28.5])	759 (22.1 [17.7–27.2])	110 (28.0 [22.6–34.1]	88 (32.0 [23.7–41.4])
12th	970 (20.8 [17.3–24.6])	878 (20.1 [16.8–23.9])	59 (29.3 [21.1–39.1])	33 (37.1 [24.8–51.3])	1,000 (21.9 [18.3–26.1])	776 (20.7 [16.9–25.2])	126 (25.2 [16.6–36.4])	98 (30.6 [23.7–38.5])
**Quit-smoking intention**								
Do not want to stop smoking cigarettes for good	425 (8.6 [7.6–9.8])	316 (6.9 [6.0–7.9])	66 (31.8 [23.6–41.3])	43 (49.3 [36.2–62.4])	317 (8.5 [7.4–9.9])	136 (4.1 [3.4–5.0])	74 (20.2 [16.2–24.9])	107 (38.8 [33.0–44.9])
Want to stop smoking cigarettes for good	355 (7.2 [6.1–8.5])	275 (5.9 [5.0–6.9])	59 (29.2 [21.7–38.0])	21 (24.8 [14.6–39.0])	253 (5.9 [4.8–7.2])	129 (3.4 [2.6–4.5])	65 (15.2 [9.6–23.1])	59 (18.6 [13.9–24.5])
Do not smoke cigarettes now	3,889 (84.2 [82.4–85.9])	3,787 (87.3 [85.7–88.6])	76 (39.1 [29.4–49.6])	26 (25.9 [16.8–37.9])	3,355 (85.6 [84.1–87.0])	2,990 (92.4 [90.6–93.9])	246 (64.6 [57.1–71.5])	119 (42.6 [37.1–48.4])
**Attempted to quit in the past 12 months**								
Did not try to quit smoking cigarettes	431 (8.5 [7.4–9.9])	340 (7.2 [6.1–8.4])	51 (23.5 [17.0–31.7])	40 (47.9 [36.2–57.9])	304 (7.7 [6.3–9.4])	136 (4.0 [3.4–4.6])	69 (19.4 [13.9–26.5])	99 (32.1 [28.2–36.3])
Tried to quit smoking cigarettes for good	631 (13.1 [11.4–14.9])	499 (11.0 [9.6–12.6])	98 (49.0 [38.3–57.8])	34 (36.7 [26.0–48.9])	481 (11.9 [10.6–13.4])	230 (6.6 [5.2–8.4])	140 (34.6 [25.9–44.6])	111 (38.4 [33.0–44.0])
Did not smoke cigarettes	3,630 (78.4 [76.1–80.1])	3,562 (81.8 [79.8–83.6])	53 (27.5 [19.7–36.9])	15 (15.4 [8.0–27.7])	3,200(80.4 [78.4–82.1])	2,950 (89.4 [87.4–91.2])	178 (46.0 [39.7–52.3])	71 (29.5 [23.6–36.2])
**Abstinence from cigarette use**								
Never tried to quit	469 (9.6 [8.3–11.1])	386 (8.4 [7.1–9.8])	45 (21.9 [15.2–30.6])	38 (48.7 [39.7–57.7])	333 (8.5 [7.2–9.9])	168 (4.8 [4.1–5.6])	73 (20.5 [15.8–26.3])	92 (32.3 [27.0–38.2])
<30-day abstinence	383 (7.7 [6.3–9.2])	285 (6.1 [4.9–7.6])	74 (34.4 [25.8–44.1])	24 (27.0 [25.8–44.1])	301 (7.2 [6.3–8.3])	158 (4.5 [3.6–5.7])	67 (15.2 [10.9–20.8])	76 (26.1 [21.6–31.2])
30-day abstinence	151 (2.9 [2.4–3.6])	132 (2.7 [2.2–3.2])	14 (8.5 [5.5–12.7])	5 (3.3 [1.2–8.5])	107 (2.8 [2.1–3.8])	47 (1.5 [0.9–2.3])	39 (9.6 [5.8–15.4])	21 (8.6 [5.0–14.2])
6-month abstinence	105 (2.1 [1.6–2.6])	82 (1.7 [1.3–2.3])	19 (9.0 [5.3–14.8])	4 (2.9 [1.0–7.8])	96 (2.1 [1.6–2.9])	53 (1.4 [1.0–2.0])	30 (6.8 [4.0–11.3])	13 (4.1 [2.2–7.8])
1-year abstinence	234 (4.7 [4.0–5.5])	207 (4.5 [3.7–5.4])	22 (10.1 [5.8–16.9])	5 (4.2 [1.3–12.2])	187 (4.6 [3.8–5.4])	123 (3.4 [2.6–4.3])	143 (13.3 [10.2–17.3])	21 (5.8 [2.9–11.2])
Never smoked cigarettes	3,350 (73.1 [70.2–75.7])	3,310 (76.6 [74.0–79.1])	27 (16.2 [10.3–24.4])	13 (14.0 [7.7–24.0])	2,954 (74.8 [72.8–76.7])	2,770 (84.5 [82.0–86.8])	129 (34.6 [28.0–41.9])	55 (23.1 [17.5–30.0])

**Table 3:** Association Between Past and Current E-Cigarette Use and Cigarette Smoking by Adolescents in the 2011 and 2013 North Carolina Youth Tobacco Surveys

Smoked Cigarettes?	E-cigarette Use, RRR (95% CI)^a^			
	2011 (n = 4,791)		2013 (n = 4,092)	
	Past Versus Never	Current Versus Never	Past Versus. Never	Current Versus Never
Never	1.00 [Reference]	1.00 [Reference]	1.00 [Reference]	1.00 [Reference]
In the past	20.95 (6.14–71.45)	4.97 (1.69–14.63)	9.66 (6.82–13.69)	9.21 (6.38–13.28)
Currently	53.17 (17.14–164.90)	43.19 (16.99–109.76)	18.68 (12.95–26.93)	46.17 (28.98–73.55)

Abbreviations: RRR: relative risk ratio; CI: confidence interval.

a Adjusted for sex, race, and school grade.

**Smoking cessation behaviors and e-cigarette use**. The number of adolescents who got tobacco cessation help was low. Among those who responded that they used any method to quit tobacco for good in the past 12 months (n = 517), the majority (n = 357; 70.1%) reported trying to quit on their own or “quit cold turkey” (Table 1). Among those who reported using cessation aids, nicotine gum (8.7%) and “help from family or friends” (8.3%) were the most common methods reported by students, followed by “attended a program” at their school or community. More than half of adolescents who reported that they used only “quitting cold turkey” (n = 317) were either current e-cigarette users (24.1%) or past e-cigarette users (33.1%). Fewer of those who reported using at least one cessation aid to help them quit than those who reported using only “quit cold turkey,” reported current e-cigarette use (15.5%) or past e-cigarette use (16.2%) (data not shown).

**Correlates of current e-cigarette use.** Table 4 shows factors significantly associated with current and past e-cigarette use in 2013. Overall, adolescents who were male, older (in higher grades), non-Hispanic white or Hispanic (compared with non-Hispanic black), had no intention to quit smoking cigarettes, or made no attempt to quit smoking cigarettes were more likely to be current e-cigarette users. Adolescents who had ever abstained from cigarette smoking for long periods and used cessation aids were less likely to be current e-cigarette users than those who made no quit attempt in the past 12 months. For example, compared with students who did not want to stop smoking for good, those who did want to stop smoking were 0.51 times more likely than nonusers to be current e-cigarette users (RRR = 0.51; 95% CI, 0.29–0.87). Current e-cigarette use was negatively associated with trying to quit smoking cigarettes in the past 12 months (RRR = 0.69; 95% CI, 0.49–0.97) and ever abstinence from cigarette smoking for 6 months (RRR = 0.42; 95% CI, 0.21–0.82) or 1 year (RRR = 0.21; 95% CI, 0.09–0.51). No association existed between reported ever abstaining for less than 6 months and current e-cigarette use. Students who reported using any kind of cessation aid to quit using tobacco in the past 12 months, including medication and family and friend support, were less likely to be current e-cigarette users (RRR = 0.46; 95% CI, 0.29–0.74).

**Table 4:** Association between past or current e-cigarette use and sociodemographics and tobacco cessation behaviors in 2013 NC Youth Tobacco Survey (n = 4,092)

Factors	RRR (95% CI)			
	Past Versus Never E-Cigarette User	*P*	Current Versus Never E-cigarette User	*P*
**Sex**				
Female	1.00 [Reference]		1.00 [Reference]	
Male	1.18 (0.90–1.54)	.22	2.84 (1.92–4.20)	<.001
**Race**				
Non-Hispanic black	1.00		1.00	
Non-Hispanic white	2.29 (1.68–3.14)	<.001	5.42 (2.83–10.36)	<.001
Non-Hispanic other	0.95 (0.42–2.19)	.91	2.06(0.86–4.98)	.10
Hispanic	1.45 (1.06–1.98)	.02.	61 (1.49–4.58)	.001
**Grade**				
9th	1.00 [Reference]		1.00 [Reference]	
10th	2.17 (1.30–3.62)	.004	1.79 (0.91–3.51)	.09
11th	2.40 (1.66–3.45)	<.001	3.07 (2.04–4.62)	<.001
12th	2.26 (1.54–3.32)	<.001	3.07 (1.89–4.99)	<.001
**Intention to quit** a				
Do not want to stop smoking cigarettes for good	1.00 [Reference]		1.00 [Reference]	
Want to stop smoking cigarettes for good	0.83 (0.50–1.36)	.45	0.51 (0.29–0.87)	.02
Do not smoke cigarettes now	0.14 (0.10–0.21)	<.001	0.05 (0.03–0.09)	<.001
**Attempted to quit in the past 12 months** a				
Did not try to quit smoking cigarettes for good	1.00 [Reference]		1.00 [Reference]	
Tried to quit smoking cigarettes for good	1.10 (0.68–1.80)	.68	0.69 (0.49–0.97)	.04
Did not smoke cigarettes	0.11 (0.07–0.17)	<.001	0.05 (0.03–0.06)	<.001
**Last abstinence from cigarette use** a				
Never tried to quit	1.00 [Reference]		1.00 [Reference]	
<30-day abstinence	0.76 (0.41–1.42)	.39	0.76 (0.51–1.15)	.19
30-day abstinence	1.54 (0.70–3.41)	.28	0.83 (0.34–2.00)	.67
6-month abstinence	1.17 (0.57–2.36)	.67	0.42 (0.21–0.82)	.01
1-year abstinence	0.88 (0.63–1.23)	.44	0.21 (0.09–0.51)	.001
Never smoked cigarettes	0.10 (0.07–0.14)	<.001	0.04 (0.03–0.07)	<.001
**Used quit aid in the last 12 months** a				
Did not attempt to quit	1.00 [Reference]		1.00 [Reference]	
Quit cold turkey only	1.72 (1.21–2.45)	.003	1.04 (0.71–1.52)	.83
Used any quit aid	0.58 (0.33-1.02)	.06	0.46 (0.29–0.74)	.002
Did not use tobacco	0.15 (0.11–0.19)	<.001	0.00 (0.00–0.00)	<.001

Abbreviations: CI, confidence interval; RRR, relative risk ratio.

a Separate models were used for each cessation-related predictor and were adjusted for sex, race, and grade.

**Correlates of past e-cigarette use.** Similar to current e-cigarette use, older, non-Hispanic white, and Hispanic students were more likely to be past e-cigarette users (Table 4). However, past e-cigarette use was not associated with adolescent intentions to quit cigarette smoking for good (RRR = 0.83; 95% CI, 0.50–1.36, *P* = .45), trying to quit smoking cigarettes in the past 12 months (RRR = 1.10; 95% CI, 0.68–1.80, *P* = .68), or ever abstinence from cigarette smoking. Students who quit cold turkey without any other cessation aids were more likely to be past e-cigarette users (RRR = 1.72; 95% CI, 1.21–2.45). Students who used cessation aids to help them quit tobacco were less likely to have used e-cigarettes in the past, but this association was moderate (RR = 0.58; 95% CI, 0.33–1.02, *P* = .06).

## Discussion

The prevalence of e-cigarette use and concurrent use of e-cigarettes and cigarettes among North Carolina adolescents increased rapidly from 2011 to 2013, consistent with national data (17,22). Given that e-cigarettes have recently become the leading form of tobacco used by US adolescents (22), the rapid increase in e-cigarette use among North Carolina cigarette “never smokers” is also concerning. Longitudinal research began to monitor the relationship between e-cigarette use and initiating cigarette smoking over time and demonstrated that e-cigarette use leads to cigarette smoking, which could potentially become the initial source of nicotine exposure and create a new generation of adolescents with nicotine addiction (23,24).

Our finding illustrated that North Carolina adolescent cigarette smokers who intended to quit or made any attempt to quit smoking cigarettes in the past 12 months were less likely to be current e-cigarette users. Such results are not surprising given that cessation is a far less commonly cited reason to use e-cigarettes among adolescents than adults (3). The relationship between e-cigarette use and intention and attempts to quit cigarette smoking were mixed across studies of US adolescents and Korean youths (17–19). The discrepant findings on quit intentions and attempts among the current and previous studies may be explained by several factors. Reasons for trying e-cigarettes may vary significantly by smoking cessation intentions and frequency of e-cigarette use, thereby making an association between cessation intentions and the use pattern of e-cigarettes nonsignificant. Research, including our study, often defines current e-cigarette use as any reported use in the past 30 days; thus, current e-cigarette users may include experimenters, unlike frequent users, who usually have different reasons for using e-cigarettes and will persist in using e-cigarettes (25). The definitions of quit intentions and attempts also vary across studies. Marked variation across studies in the measurement of adolescent e-cigarette use makes results difficult to interpret (26). Future research needs to use consistent and validated measures to assess quit intentions and attempts and to examine reasons for using e-cigarette by e-cigarette use frequency.

Similar to the findings of quit intentions and quit attempts, adolescent cigarette smokers who had ever abstained from cigarette smoking for 6 months or more were less likely to be current e-cigarette users. Future research should examine adolescents’ reasons for using cigarettes to determine whether they use e-cigarettes to quit smoking cigarettes, whether they did not want to quit smoking entirely but sought a healthier alternative, or whether they simply experimented or used e-cigarettes for recreational reasons rather than for cessation reasons. Longitudinal research is needed to determine the causal relationship between e-cigarette use and cessation outcomes by tracking the patterns of cessation behaviors and e-cigarette use and examining reasons for using e-cigarettes over time.

The majority of adolescent tobacco users reported that they either had tried to quit cold turkey or did not try to quit at all in the past 12 months. These adolescents may have felt invulnerable to health-threatening behaviors, felt optimistic about their chances of avoiding harm compared with adults’ chances, believed they were less addicted to smoking, and believed quitting would be easy (27,28). Our results also found that adolescents who reported they had used any cessation aids were less likely to be current e-cigarette users; on the other hand, those adolescents who tried quitting on their own were more likely to have used e-cigarettes in the past. It is unclear whether these adolescents experimented with e‑cigarettes out of curiosity, or used e-cigarettes as a cessation aid or a healthier alternative. Future research should understand the role of e-cigarette use in adolescents’ cessation behaviors by examining adolescents’ reasons for using e-cigarettes and distinguish between e-cigarette motivated users (eg, quitting smoking as a goal-oriented reason) and e-cigarette experimenters (eg, curiosity as a nongoal-oriented reason) (9).

Additional limitations of this study should be noted. The limited terminology of the question on e-cigarette use may underestimate e-cigarette use because it does not include terms such as “vapes,” which are commonly used by adolescents (29). The lack of a specific time frame for the measure of length of last abstinence limits our ability to examine relationships with current e-cigarette use. Our findings may not generalize to adolescents in populations other than North Carolina high school students; however, our results are similar to national results for adolescent tobacco use in the United States. Finally, because NCYTS uses a cross-sectional design, causal relationships cannot be determined, but many associations are consistent with previous cross-sectional data, providing important directions for future longitudinal research.

Our findings about rising dual use of e-cigarettes and cigarettes, along with associations between e-cigarette use and lower cessation intention and behaviors, have implications for public health practice and cessation clinic services. Our research supports FDA’s recent announcement to extend its authority to regulate e-cigarettes as tobacco products, which includes prohibiting unsubstantiated cessation aid claims and restricting the sale of e-cigarettes to adolescents under age 18 (30). The American Academy of Pediatrics called for stronger regulation of e-cigarettes, urged pediatricians to offer tobacco cessation counseling and FDA-approved treatments appropriate to an adolescent’s level of addiction and readiness to change, and disapproved e-cigarette use as a recommended treatment product for tobacco dependence (13). Messages about negative effects of e-cigarette use, including the severity and rapid development of nicotine neurotoxin and addiction, on adolescents’ health should be part of this counseling and youth tobacco control media campaigns as well.

## Post-Test Information

To obtain credit, you should first read the journal article. After reading the article, you should be able to answer the following, related, multiple-choice questions. To complete the questions (with a minimum 75% passing score) and earn continuing medical education (CME) credit, please go to http://www.medscape.org/journal/pcd. Credit cannot be obtained for tests completed on paper, although you may use the worksheet below to keep a record of your answers. You must be a registered user on Medscape.org. If you are not registered on Medscape.org, please click on the "Register" link on the right hand side of the website to register. Only one answer is correct for each question. Once you successfully answer all post-test questions you will be able to view and/or print your certificate. For questions regarding the content of this activity, contact the accredited provider, CME@medscape.net. For technical assistance, contact CME@webmd.net. American Medical Association’s Physician’s Recognition Award (AMA PRA) credits are accepted in the US as evidence of participation in CME activities. For further information on this award, please refer to http://www.ama-assn.org/ama/pub/about-ama/awards/ama-physicians-recognition-award.page. The AMA has determined that physicians not licensed in the US who participate in this CME activity are eligible for ***AMA PRA Category 1 Credits™***. Through agreements that the AMA has made with agencies in some countries, AMA PRA credit may be acceptable as evidence of participation in CME activities. If you are not licensed in the US, please complete the questions online, print the AMA PRA CME credit certificate and present it to your national medical association for review.

## Post-Test Questions

### Study Title: Electronic Cigarette Use Among High School Students and Its Association With Cigarette Use And Smoking Cessation, North Carolina Youth Tobacco Surveys, 2011 and 2013

#### CME Questions

1. You are performing a well-child examination on a 16-year-old girl, who has no complaints today. You ask her about using tobacco products, and she reports that she has used both cigarettes and electronic cigarettes (e-cigarettes) in the past 6 months. According to the current study by Huang and colleagues, which of the following statements regarding trends in the use of cigarettes and e-cigarettes between 2011 and 2013 is ***most*** accurate?
  - The rate of e-cigarette use increased, but regular cigarette use decreased
  - The rate of regular cigarette use increased, but e-cigarette use decreased
  - The rate of e-cigarette and regular cigarette use both decreased
  - The rate of e-cigarette and regular cigarette use both increased
2. You ask the patient about previous attempts to quit tobacco products. What was the ***most*** commonly cited strategy for quitting by adolescents in the current study?
  - "Cold turkey"
  - Nicotine replacement drugs
  - Peer support groups
  - Other medications
3. The patient defends her use of e-cigarettes by telling you that "everyone is smoking them!" Which of the following variables correlated ***most*** with the current use of e-cigarettes in the current study?
  - Female sex
  - Younger age
  - Non-Hispanic Black race
  - Failure to use cessation aids to quit smoking
4. You discuss quitting both regular cigarettes and e-cigarettes with the patient. What should you consider regarding correlations between quitting regular cigarettes and e-cigarette use in the current study?
  - Most current users of e-cigarettes had plans to quit regular cigarettes
  - Current e-cigarette use was negatively associated with quit attempts in the past 12 months
  - Past e-cigarette use was strongly associated with a desire to quit smoking for good
  - Past e-cigarette use was associated with a higher rate of trying to quit in the past 12 months

**Table:** Evaluation

**1. The activity supported the learning objectives.**				
**Strongly Disagree**				**Strongly Agree**
1	2	3	4	5
**2. The material was organized clearly for learning to occur.**				
**Strongly Disagree**				**Strongly Agree**
1	2	3	4	5
**3. The content learned from this activity will impact my practice.**				
**Strongly Disagree**	** **	** **	** **	**Strongly Agree**
1	2	3	4	5
**4. The activity was presented objectively and free of commercial bias.**				
**Strongly Disagree**				**Strongly Agree**
1	2	3	4	5

## References

[1] Grana RA , Ling PM . “Smoking revolution”: a content analysis of electronic cigarette retail websites. Am J Prev Med 2014;46(4):395–403. 10.1016/j.amepre.2013.12.010 24650842 PMC3989286

[R2] Richardson A , Ganz O , Stalgaitis C , Abrams D , Vallone D . Noncombustible tobacco product advertising: how companies are selling the new face of tobacco. Nicotine Tob Res 2014;16(5):606–14. 10.1093/ntr/ntt200 24379146

[3] Kong G , Morean ME , Cavallo DA , Camenga DR , Krishnan-Sarin S . Reasons for electronic cigarette experimentation and discontinuation among adolescents and young adults. Nicotine Tob Res 2015;17(7):847–54. 10.1093/ntr/ntu257 25481917 PMC4674436

[4] Choi K , Forster J . Characteristics associated with awareness, perceptions, and use of electronic nicotine delivery systems among young US Midwestern adults. Am J Public Health 2013;103(3):556–61. 10.2105/AJPH.2012.300947 23327246 PMC3567225

[5] Dockrell M , Morrison R , Bauld L , McNeill A . E-cigarettes: prevalence and attitudes in Great Britain. Nicotine Tob Res 2013;15(10):1737–44. 10.1093/ntr/ntt057 23703732 PMC3768337

[6] Adkison SE , O’Connor RJ , Bansal-Travers M , Hyland A , Borland R , Yong H-H , Electronic nicotine delivery systems: international tobacco control four-country survey. Am J Prev Med 2013;44(3):207–15. 10.1016/j.amepre.2012.10.018 23415116 PMC3627474

[7] Etter J-F , Bullen C . Electronic cigarette: users profile, utilization, satisfaction and perceived efficacy. Addiction 2011;106(11):2017–28. 10.1111/j.1360-0443.2011.03505.x 21592253

[8] Vickerman KA , Carpenter KM , Altman T , Nash CM , Zbikowski SM . Use of electronic cigarettes among state tobacco cessation quitline callers. Nicotine Tob Res 2013;15(10):1787–91. 10.1093/ntr/ntt061 23658395

[9] Pepper JK , Ribisl KM , Emery SL , Brewer NT . Reasons for starting and stopping electronic cigarette use. Int J Environ Res Public Health 2014;11(10):10345–61. 10.3390/ijerph111010345 25286168 PMC4210982

[10] Rahman MA , Hann N , Wilson A , Worrall-Carter L . Electronic cigarettes: patterns of use, health effects, use in smoking cessation and regulatory issues. Tob Induc Dis 2014;12(1):21. 10.1186/1617-9625-12-21 25745382 PMC4350653

[11] Slotkin TA . Cholinergic systems in brain development and disruption by neurotoxicants: nicotine, environmental tobacco smoke, organophosphates. Toxicol Appl Pharmacol 2004;198(2):132–51. 10.1016/j.taap.2003.06.001 15236950

[12] Walley SC , Jenssen BP ; Section on Tobacco Control. Electronic nicotine delivery systems. Pediatrics 2015;136(5):1018–26. 10.1542/peds.2015-3222 26504128

[13] McRobbie H , Bullen C , Hartmann-Boyce J , Hajek P . Electronic cigarettes for smoking cessation and reduction. Cochrane Database Syst Rev 2014;12(12):CD010216. 25515689 10.1002/14651858.CD010216.pub2

[14] DiFranza JR , Savageau JA , Fletcher K , O’Loughlin J , Pbert L , Ockene JK , Symptoms of tobacco dependence after brief intermittent use: the Development and Assessment of Nicotine Dependence in Youth-2 study. Arch Pediatr Adolesc Med 2007;161(7):704–10. 10.1001/archpedi.161.7.704 17606835

[15] Mermelstein R . Teen smoking cessation. Tob Control 2003;12(90001, Suppl 1):i25–34. 10.1136/tc.12.suppl_1.i25 12773783 PMC1766088

[16] Dierker L , Hedeker D , Rose J , Selya A , Mermelstein R . Early emerging nicotine dependence symptoms in adolescence predict daily smoking in young adulthood. Drug Alcohol Depend 2015;151:267–71. 10.1016/j.drugalcdep.2015.03.009 25840749 PMC4447570

[17] Dutra LM , Glantz SA . Electronic cigarettes and conventional cigarette use among U.S. adolescents: a cross-sectional study. JAMA Pediatr 2014;168(7):610–7. 10.1001/jamapediatrics.2013.5488 24604023 PMC4142115

[18] Park J-Y , Seo D-C , Lin H-C . E-Cigarette use and intention to initiate or quit smoking among US youths. Am J Public Health 2016;106(4):672–8. 10.2105/AJPH.2015.302994 26794178 PMC4986055

[19] Lee S , Grana RA , Glantz SA . Electronic cigarette use among Korean adolescents: a cross-sectional study of market penetration, dual use, and relationship to quit attempts and former smoking. J Adolesc Health 2014;54(6):684–90. 10.1016/j.jadohealth.2013.11.003 24274973 PMC4031306

[20] Simon P , Kong G , Cavallo DA , Krishnan-Sarin S . Update of adolescent smoking cessation interventions: 2009–2014. Curr Addict Rep 2015;2(1):15–23. 10.1007/s40429-015-0040-4 26295017 PMC4540362

[21] Proescholdbell SK , Summerlin-Long SK , Goldstein AO . Declining tobacco use among North Carolina middle and high school students: 1999–2007. N C Med J 2009;70(3):205–12. 19653602

[22] Arrazola RA , Singh T , Corey CG , Husten CG , Neff LJ , Apelberg BJ , ; Centers for Disease Control and Prevention (CDC). Tobacco use among middle and high school students — United States, 2011–2014. MMWR Morb Mortal Wkly Rep 2015;64(14):381–5. 25879896 PMC5779546

[23] Primack BA , Soneji S , Stoolmiller M , Fine MJ , Sargent JD . Progression to traditional cigarette smoking after electronic cigarette use among US adolescents and young adults. JAMA Pediatr 2015;169(11):1018–23. 10.1001/jamapediatrics.2015.1742 26348249 PMC4800740

[24] Leventhal AM , Strong DR , Kirkpatrick MG , Unger JB , Sussman S , Riggs NR , Association of electronic cigarette use with initiation of combustible tobacco product smoking in early adolescence. JAMA 2015;314(7):700–7. 10.1001/jama.2015.8950 26284721 PMC4771179

[25] Amato MS , Boyle RG , Levy D . How to define e-cigarette prevalence? Finding clues in the use frequency distribution. Tob Control 2016;25(e1, e1):e24–9. 10.1136/tobaccocontrol-2015-052236 26085124 PMC4683118

[26] Echevarria C , Sinha IP . Heterogeneity in the measurement and reporting of outcomes in studies of electronic cigarette use in adolescents: a systematic analysis of observational studies. Tob Control 2016;tobaccocontrol-2015-052881. Published online Apr 29, 2016 10.1136/tobaccocontrol-2015-052881 27129981

[27] Amos A , Wiltshire S , Haw S , McNeill A . Ambivalence and uncertainty: experiences of and attitudes towards addiction and smoking cessation in the mid-to-late teens. Health Educ Res 2006;21(2):181–91. 10.1093/her/cyh054 16107488

[28] Cohn LD , Macfarlane S , Yanez C , Imai WK . Risk-perception: differences between adolescents and adults. Health Psychol 1995;14(3):217–22. 10.1037/0278-6133.14.3.217 7641662

[29] Wagoner KG , Cornacchione J , Wiseman KD , Teal R , Moracco KE , Sutfin EL . E-cigarettes, hookah pens and vapes: adolescent and young adult perceptions of electronic nicotine delivery systems. Nicotine Tob Res;2016. Epub Mar 30, 2016. 10.1093/ntr/ntw095 27029821 PMC5016844

[30] Food and Drug Administration. FDA takes significant steps to protect Americans from dangers of tobacco through new regulation. Accessed May 5, 2016. http://www.fda.gov/NewsEvents/Newsroom/PressAnnouncements/ucm499234.htm.

